# Learners' perspectives on the prevention and management of pregnancy school dropout: a Namibian case

**DOI:** 10.3389/fsoc.2023.1182163

**Published:** 2023-06-22

**Authors:** Dorthea Nanghali Etuwete Shiningayamwe

**Affiliations:** ^1^Sustainability Research, Tokyo University of Foreign Studies, Fuchu, Japan; ^2^Department of Academic Affairs-Contemporary Social Issues, University of Namibia, Windhoek, Namibia

**Keywords:** learner pregnancy, school dropouts, prevention, management, Namibia

## Abstract

**Background:**

Namibia has had a problem with the high rate of learner pregnancy and school dropout for many years, despite implementing education sector policy on preventing and managing learner pregnancy. This study aimed to explore the perspectives of school-going learners in Namibia regarding the factors contributing to learner pregnancy and school dropout and propose interventions to address them.

**Methods:**

This qualitative research employed interpretative phenomenological data analysis, with seventeen individual and ten focus group interviews involving 63 school-going learners: adolescents, pregnant learners, and learner parents.

**Results and findings:**

Emerging factors driving learner pregnancy and school dropout in rural Namibian schools include older men and cattle herders preying on young girls, long school holidays, the proximity of alcohol sites near school premises, and age restrictions after maternity leave. The learners proposed interventions include prohibiting learners' access to alcohol establishments, strengthening collaborations between stakeholders, sensitizing girls and cattle herders, and ongoing advocacy efforts. Findings indicate community hostility, lack of infrastructure and resources, and learner unawareness. It is essential to mitigate community hostility and raise awareness. Incorporating the perspectives of learners in policy interventions remains crucial for effectively addressing the high rates of learner pregnancy and school dropout in rural Namibian schools.

## 1. Introduction

In Africa, teenage pregnancy remains a significant barrier to achieving gender equality and a leading factor in girls dropping out of school before completing primary education cycles (UNESCO, 2017, 2018; World Vision, 2020). The sub-Saharan African governments introduced comprehensive sexuality education (CSE) into school curricula and implemented laws allowing pregnant and learner-parents to complete their education (Mutua et al., 2019; Zulu et al., 2019; Human Rights Watch, 2021; Chinkondenji, 2022). However, measures have been inadequate, leading to persistent educational barriers for girls due to pregnancy and school dropout in the continent (Zuilkowski et al., 2019; Zulu et al., 2019; Adekola and Mavhandu-Mudzusi, 2023).

Namibia is not an exception. The Namibian government introduced the Education Sector Policy on the Prevention and Management of Learner Pregnancy (ESPPMLP) to decrease the number of learners becoming pregnant and increase the number of learner-parents completing education (Ministry of Education, 2012). Despite the existence of the ESPPMLP, Education Management Information System (EMIS) Reports showed that the number of pregnancy-related school dropouts increased from 1,560 in 2019 to 2,291 in 2020 and 3,658 in 2021 (Ministry of Education, 2021). The cases include primary school learners as young as 11, leading to public outcry and media coverage in the country (The Namibian, 2021, 2022a,b,c). According to the Ministry of Education (2012), the accepted definition of a learner-parent encompasses learners who become a parent while attending school. Learner pregnancy and school dropout refer to an incident where a learner dropped out of school due to pregnancy. A learner-mother and father are those who become mothers or fathers while attending school.

This study argues that despite efforts to democratise the decision and policy-making process, the views and experiences of the learners remain marginalised (Nyariro, 2018). Their experiences are complexly intertwined with their social context, opposing how others discuss them within communities and not aligning with policy provisions (Ruzibiza, 2021; Adekola and Mavhandu-Mudzusi, 2023). Therefore, including learners' voices in developing effective mitigation strategies and policy guidelines are essential as they possess first-hand experience and insight. In addition, the perspectives of learners enable policymakers, educators, and other stakeholders to understand the underlying obstacles related to learner pregnancy and school dropout, which could be easier to allocate appropriate resources and establish tailored support systems that address their needs (Adekola and Mavhandu-Mudzusi, 2023).

Previous studies in Namibia widely researched factors contributing to learners' pregnancy and school dropouts and discovered factors, such as low contraceptive use, early sexual debut, poverty, lack of parental involvement, cultural norms, and poor school performance (Burton et al., 2011; Eloundou-Enyegue and Shirley, 2011; Nekongo-Nielsen and Mbukusa, 2013; David et al., 2017; Maemeko et al., 2018; Indongo, 2020; Mogotsi and Mwetulundila, 2020). However, their findings have neither been related to the implementation of the Namibia ESPPMLP nor underscore the learner's perspectives. This is substantial to document evidence-based views from the learners that can be applied to improve the implementation of the ESPPMLP. Therefore, this study aims to investigate learners' perspectives on preventing and managing learner pregnancy and school dropout concerning the ESPPMLP in rural Namibian schools.

## 2. Background and context of the study

### 2.1. The development of the ESPPMLP in Namibia

Before Namibia's independence on March 21, 1990, pregnant learners in Namibia were often expelled from school as a form of punishment, exacerbating the inequality in education (Burton et al., 2011). However, after gaining independence, the parents and community members demanded the reintegration of learner-parents and pregnant learners into schools. Namibia recognised the need to address gender inequalities in education and thus adopted policies to prevent and manage learner pregnancy and school dropout (Legal Assistance Centre, 2015).

The Education Sector Policy on the Prevention and Management of Learner Pregnancy has two main components: prevention and management. Prevention aims to provide age-appropriate life skills programs to Namibia's primary and secondary school learners through compulsory but non-promotional subjects of life skills covering topics such as CSE, abstinence, sexual risks, contraception, gender sensitivity, child abuse, and sexual violence awareness. The management takes over when prevention efforts fail and a learner becomes pregnant or impregnates a fellow learner. Learner-fathers can remain in school without having to take leave. The pregnant learners can continue attending school until 4 weeks before giving birth or take leave at any stage of pregnancy. After giving birth, learner-mothers may choose to return to school, extend their leave to 1 year, or transfer to another school. Schools reserve learner-mothers' spaces provided learners update their intended return date. The parents or caregivers must engage with the school and sign a statement to ensure someone will care for the infant while the mother attends classes.

Besides, management ensures that the learner-mother, father, and learner must receive psychosocial support and counsel from a trusted adult on pregnancy, sexual and reproductive health, and other social issues in school settings. The policy emphasises confidentiality in school settings. It addresses the stigma and discrimination that pregnant, or learner-parents face from teachers and fellow learners through disciplinary measures. In addition, the ESPPMLP mandates the involvement of relevant stakeholders, such as parents, NGOs, and four-line ministries, including the Ministry of Gender Equality and Child Welfare, which must assign social workers for learners' counselling. The Ministry of Health and Social Services provides adolescent-friendly health services through mobile services in school settings, offering pre-and post-natal care, HIV counselling, and related services. The Ministry of Safety and Security Services offers prompt and sensitive social services and investigates cases of rape and other crimes. The ESPPMLP prohibits teachers from engaging in sexual relationships with learners. The teacher faces suspension from the profession, regardless of whether it results in pregnancy.

### 2.2. The Namibian status of learner pregnancy and school dropout

Despite the investments in addressing the prevention and management of learner pregnancy and school dropout, Namibia continues to face a significant challenge with teenage pregnancy. With a young population, the country has the highest adolescent birth rate of 82 births per 1,000 girls aged 15–19 years, double the global average of 44 (Namibia Statistics Agency, 2014). Teenage pregnancy is one of the leading causes of girls dropping out of upper primary school and failing to graduate high school on time (Ministry of Education, 2020). Rural schools experience learner pregnancy dropouts at three times the rate of urban schools (UNESCO, 2018). Pregnant learners are part of the 13 groups of marginalised children who cannot complete their education in Namibia (Pearson and Van Der Berg, 2015). Between 2018 and 2021, Namibia had 56,300 cases of teenage pregnancy, exceeding the number of pupils qualified for university education, which was only 37,480 during the same period. In the first 2 months of 2022, there were over 2,400 reported cases of teenage pregnancies nationwide (The Namibian, 2022c). Statistics from the EMIS report for the 2016 to 2020 academic years indicate that Namibia had over 10,000 learners dropping school due to learner pregnancies (Ministry of Education, 2016). This number adds to the staggering figure of over 50,000 learners who drop out of school annually in Namibia (Julius and Amupanda, 2017).

Studies have shown that in addition to losing educational opportunities, pregnancy-related school dropouts face limited prospects, such as a lack of skills, income, food, shelter, access to better health, and face difficulties in entering the labour market later in life (David et al., 2017; Julius and Amupanda, 2017; Legal Assistance Centre, 2017; Ministry of Sport, Youth and National Services, 2020). Learners who drop out of school migrate from rural to urban areas for better living conditions. Women may turn to criminal activities, such as illegal drug dealing, alcohol abuse, sex work, early marriages, or transactional relationships with multiple sexual partners, to cope with their lack of education or unemployment (Legal Assistance Centre, 2017). If this trend is left unchecked, it could significantly strain the government's ability to allocate resources and services such as housing for unemployed individuals, food, sanitation, and access to primary schools and health. This could hinder national policies that target to reduce poverty, gender inequality, unemployment, and other socio-economic issues facing Namibia.

### 2.3. Literature

The previous literature highlights that pregnant and learner-parents face stigma and discrimination from school teachers, and peers disgust them negatively as “Life Skills teacher's learners” (Legal Assistance Centre, 2017; UNESCO, 2018). Nekongo-Nielsen and Mbukusa (2013) stress the importance of establishing a secure, friendly, and encouraging learning environment in schools and recording the perpetrators' identity to address the increase in pregnancy and school dropout rates. Burton et al. (2011) and the Legal Assistance Centre (2017) reveal that sexual harassment and abuse frequently occur in Namibian schools, with many unreported cases. Pearson and Van Der Berg (2015) and UNESCO (2018) report that Namibian teachers have been known to exploit their positions of authority by engaging in sexual relationships with minor learners. It is worth noting that no law punishes those who engage in sexual affairs with learners except school teachers. Studies conducted by Burton et al. (2011) and Pearson and Van Der Berg (2015) shed light that walking long distances to and from school in Namibia poses a risk of youth victimisation to social evils, making it challenging for every child in the country to attend school.

Additionally, a study on comprehensive sexuality education (CSE) scaling up in practise from Eastern and Southern Africa by UNESCO (2017) elucidated that the Namibian curricula's breadth of CSE content is insufficient. The same study cited a lack of specialised CSE programs at the pre-set level, which could hinder learners from being adequately capacitated to make informed decisions about sexual reproductive issues. Mogotsi and Mwetulundila (2020) raised concerns over the neglect of father-learners' needs in sexual and reproductive programs within the community, underscoring the need for educational interventions to recognise and target male youth to promote positive development. UNESCO (2018) highlighted a lack of formal linkages between schools, health services, and relevant Namibia stakeholders such as parents, religious institutions, and NGOs. In their study, UNESCO highlighted that although learners receive information on Sexual Reproductive Health (SRH) and where to access services, there are weak referral systems to other service providers and limited provision due to resistance from school principals, teachers, parents, and community sensitivities. To address these issues, it is necessary to establish and communicate a minimum CSE package for schools, including referrals (Lukolo and van Dyk, 2014; UNESCO, 2017).

According to World Vision (2020), several countries have implemented strategies to prevent and manage learner pregnancy and school dropout. For example, Gabon has nurseries and childhood centres near schools. Cape Verde and Senegal provide young mothers with exceptional accommodations, such as breastfeeding breaks or time off for infant care. In Ghana, communities have used social accountability to address the issue through civic education and monitoring of national policies. They also have ongoing public awareness campaigns and targeted back-to-school initiatives for pregnant learners through television, radio, and social media advocacy messages. In the same study, World Vision (2020) recommended establishing sexual violence reporting mechanisms through child-friendly, community-based monitoring and responses to ensure the return of vulnerable learner parents at greater risk of not returning to school, collaboration with community members, local politicians, community chiefs, teachers, caregivers, and social workers. Following up, learning about their situations, and facilitating their return to school is also essential. One school community in Ghana successfully used this approach, dropping learner pregnancy school dropouts from seven in 2015 to three in 2016 and none in 2017 (World Vision, 2020). Maharaj (2022) suggested a paradigm shift to support grants through conditional cash transfers as a double-edged sword in reducing adolescent pregnancy and school dropout. This was confirmed in Colombia, where pregnant and learner-parents received a subsidy if they attended school, completed their school year, and enrolled in the following years.

While existing literature has explored diverse perspectives on preventing and managing learner pregnancy and school dropout from various stakeholders, such as social determinants, demographic data, teachers, community voices, and male youth participants, the lack of learners' perspectives in previous studies limits understanding of how well the ESPPMLP works and what improvements can be made. Therefore, this study investigates learners' perspectives on preventing and managing pregnancy and school dropout in selected rural Namibian schools regarding the ESPPMLP for its improvements.

## 3. Research methods

### 3.1. Design

The study utilised a phenomenological research design to investigate and elucidate the perspectives of learners on the prevention and management of pregnancy and school dropout in selected rural Namibian schools. Employing a phenomenological approach allows researchers to delve deeper into understanding the essence of the participant's experiences and the contextual factors contributing to those experiences (Cresswell and Cresswell, 2018; Cresswell and Poth, 2018). During the research process, the participants were asked probing questions to elicit their insights and experiences regarding preventing and managing learner pregnancy and school dropout within their schools, communities, families, and social circles. The researcher attentively listened to and documented the participants' recorded narratives and firsthand encounters related to the investigated issue. In addition, the researcher employed a case study: a qualitative approach that involves exploring a real-life, bounded system through in-depth data collection using various methods such as observation, interviews, documents, and reports (Cresswell and Poth, 2018). The rationale for choosing a case study design focused on accessing multiple units, specifically different schools from the same region, each with varying proportions of learner pregnancy and school dropout cases. By considering the geographical setup and socio-economic dynamics, this study aimed to capture different perspectives of learners from different schools on the same issue (Cresswell and Poth, 2018).

### 3.2. Site

The study occurred in one of Namibia's 14 regions, located in the far northern area of Namibia, which borders Angola Cunene province. The region is typically rural, and most schools are in rural zones. According to the 2020 EMIS report, the region had 270 schools with 110,127 learners, of which 49.7% were female students (Ministry of Education, 2020). The region recorded the highest number of pregnant teenagers in the country from 2010 to 2022, with 23,700, at a prevalence rate of 11.6%, linked to the lowest rate of contraceptive use among teenage girls, with 13.6% (Indongo, 2020; The Namibian, 2022b). The region had high learner pregnancies and school dropout cases in three academic years, 2017, 2018, and 2019 (Ministry of Education, 2017, 2018, 2019).

### 3.3. Sampling

The data presented in this study targeted school learners in selected primary and secondary schools located within a rural region in northern Namibia. We utilised purposive sampling to select schools and individuals that can purposefully inform an understanding of the research problem (Cresswell and Poth, 2018). The participating schools were determined by the number of educational circuits in the region [targeting one school per circuit (the region has 10 circuits)] and by intentionally selecting any school-going learner who demonstrated features of interest and was affected by learner pregnancy. We purposely selected schools with a consistent record of high learners' pregnancy school dropout incidents in three academic years (2017–2019). The inclusion criteria for participants were as follows: participants had to attend schools in the rural areas of the undisclosed region in Namibia at the time of data collection. The school of attendance had to consistently record learner pregnancy and school dropout incidents over 3 years (2017–2019). Other inclusion criteria involved communicating in Oshiwambo or English, being ready for audio recording, providing informed consent, and obtaining parental consent. We targeted learners between 13 and 18 years. However, we found learners aged 23 years attending the same schools within the 13–18 age range, and we included them in the study as they expressed willingness to share their experiences. We covered 63 participants in total, as presented in the table below.

### 3.4. Data collection

The researcher conducted 17 individual and 10 focus group interviews between August and October 2021 with 63 learners. The study followed a set of semi-structured interviews, both for the individual interview guide and the focus group interview guide. Following the interview guidelines by Kvale (1996), Qu and Dumay (2011) cited in Adekola and Mavhandu-Mudzusi (2023), the researcher asked central questions such as “Based on your experiences and perspectives, please tell me the factors that contribute to learner pregnancy at your school and around your community? Why do pregnant and learner parents tend to drop out of school?” This was followed by probing questions for rich and comprehensive data.

The interviews were conducted across three categories: adolescents, pregnant learners, and learner-parents. Despite the distinctions, the questions remained broadly consistent. Individual interviews with pregnant learners and learner-parents were conducted in private settings, such as a school storeroom, to ensure privacy and address the sensitivity of the matters discussed. Considering the busy class schedules, we sometimes followed the pregnant learners and learner-parents at their homes after school hours while maintaining privacy during interviews.

Conversely, interviews with adolescent learners were conducted through focus group discussions held in open school areas, such as under a tree or in open classrooms. The focus group discussions took place after school or during break times. Each interview session lasted ~1 h and 30 min; recordings were made for transcription. All interviews were conducted in both English and Oshiwambo languages.

After each interview session, we provided written notes to each participant, allowing them to share any additional information about the topic confidentially. This approach, aligned with Cresswell and Cresswell (2018), recognised that not all individuals are equally articulate or express their perspectives during interviews. We further allowed participants to write their notes and discreetly pass them through the researcher's open car window on the school premises to ensure anonymity. This method facilitated a sense of security and freedom for participants to share their experiences without fear of identification.

### 3.5. Data analysis

The data analysis process began during the interview sessions, where the researcher noted emerging words from the voice recordings. After the data collection phase, the researcher transcribed the audio-recorded data using the interpretative phenomenological analysis data framework. This approach allows the participants to express their lived experiences and stories as they see fit without any distortion and prosecution (Alase, 2017). The researcher carefully listened to the recorded audio, transcribed some audio from Oshiwambo to English, and simultaneously used the observation's non-verbal cues, reflections, and thoughts related to the participant's narratives. This iterative process aimed to capture and preserve the richness of the participants' lived experiences. The identified themes were then organised into superordinate themes, ensuring that the analysis reflected the depth and complexity of the data. A comparative analysis of the emerging themes was conducted, creating a comprehensive table of themes. This table comprised two superordinate themes, several sub-themes, and relevant participant quotes. Including participants' own words helped maintain the authenticity and credibility of the findings. To enhance trustworthiness, the identified themes were carefully aligned with the goals and directives of the ESPPMLP, ensuring coherence between the research findings and the study's overall purpose. Additional reviews were carried out to identify any potential overlaps or contradictions within the themes, further strengthening the reliability of the analysis.

### 3.6. Ethics

Permission to conduct the research was obtained from the Tokyo University of Foreign Studies where the researcher is a student pursuing a Ph.D. This was followed by a second permission acquired through an authorisation letter from the Namibia Ministry of Education's Executive Director at the Head Offices. At the regional level, the Education Director in the region also signed off the study permission letter. These letters were then presented to the school principal for their approval. This procedure ensured that all administrative parties granted their consent for the research. Since the researcher has no prior relationship with the participants, the life skills teacher introduced and announced the researcher at the morning assembly for interested participants to volunteer and convene to meet after school. We thoroughly explained the study instructions to all participants and ensured their understanding that it was voluntary. Any participants could withdraw at any point.

To ensure that participants were well-informed about the objectives, procedures, and expectations, an orientation session about the study was conducted 1 week before every school's scheduled data collection date, reiterating that their participation was voluntary and had the freedom to withdraw at any point, even with parental consent. Given that some participants were minors, we handed individual and parental consent for their participation. Prior to data collection, all participants submitted their consent forms, including parental consent. To maintain anonymity and confidentiality (Sim and Waterfield, 2019), participants were instructed not to use their real names and assured that all information would be confidential.

## 4. Results

This study aims to investigate learners' perspectives on preventing and managing learner pregnancy and school dropout in relation to the ESPPMLP in rural Namibian schools.

### 4.1. The learners' perspectives on the causes of learner pregnancy and school dropout

This theme presents the perspectives of learners based on their experiences on the factors that contribute to learner pregnancy and barriers that lead to school dropout among pregnant learners and learner parents in the schools and communities.

#### 4.1.1. Elder men, cattle herders, and police officers preying on young girls, schools

The interviewees revealed that grown men, including those in positions of authority, are preying on vulnerable girls as young as 11 years old by luring them with little gifts. Fellow learners (father-learners) were cited in rare cases. The men are not just random strangers but include people in positions of authority and professional men who approach young for sexual relationships.

In addition, when asked why cases are high, many learners, with disdain and blame, cited, “*The cattle herders are sleeping with our young girls. Blame it on them*.” The participants expressed that men from a neighbouring country are employed as cattle herders in their communities. These men are from a lower socio-economic and educational background and seek employment opportunities. These cattle herders view the schoolgirls as easy targets for sexual relationships. They often stalk the girls in Savannah areas and on their way to and from school. They entice them with small gifts and money, making it easier to coerce the girls into sexual favours. “*Many of my friends have fallen pregnant due to sexual relationships with male cattle herders. The cattle herders entice girls with gifts such as radios with USBs to play music. I also slept with a cattle herder but did not fall pregnant.” [Maya, 15 years Female]*

Some participants reported that their schools are near roadblocks and police stations, where police officers frequently inspect traffic and transport goods. As a result, learners pass by these areas on their way to and from school. This exposes them to police officers to coerce them into sexual relationships. The learners expressed discomfort and fear at these experiences, feeling sexually intimidated and powerless to change their route to school.

“*Our school is located near a police roadblock, and as a girl, you can never walk alone without being called by a police officer. Sometimes it is scary, and you start to think you did something wrong. Once you go there, the police officer asks for your cell number and if they can see you later. I am worried that the police officers are interested in young girls. If I report the officer's friend, would he protect his friend?” [Martha, 18 years, Female]*

#### 4.1.2. No knowledge that sexual harassment and statutory rape are crimes

The study revealed that although the ESPPMLP aims to educate learners on sexual violence awareness and empower them to report indecent assaults, the interviewed learners demonstrated a lack of knowledge that statutory rape and sexual harassment perpetrated against a minor violate the law. When asked whether they were aware that it is criminal law, many learners responded with a “No,” indicating a lack of familiarity with this term and its legal implications. Similarly, one of the anonymously written notes from a learner-parents who encountered rape sought guidance from the researcher on responding to these traumatic experiences.

“*When I was 15 years old in 2020, I got involved in a case rape. My friend's brother raped me. So, I disclosed it to my life skills teacher on a Monday since it happened on a Friday. This made me very emotional and shy to go out of the house. However, thanks to God, I was not pregnant. What can we do now?” [Fiina, 17 years, female]*

#### 4.1.3. “Shebeen and cuca shop” alcohol sites within schools and threats of sexual coercion

Participants revealed that the proximity of alcohol sites near schools poses a significant danger to learners, especially at a young age, as their peers tend to pass by these alcohol sites on their way home near the school premises, leading to dangerous situations such as hanging out with strange men at bars and receiving free drinks while still in school uniforms. They believed that such sites expose many learners to the earlier taste of alcohol, with girls particularly vulnerable to the social evils associated with alcohol consumption. Participants mentioned that even young boys are at risk of being propositioned by older women in exchange for alcohol, increasing the risk of unwanted pregnancies and other negative consequences.

Some female learners reported becoming pregnant unwillingly due to persistent sexual coercion and persuasion from older male perpetrators who relentlessly pursued them until they gave in to their advances. Also, sexual coercion occurs at water points and after-school activities such as sports, church, and other public gatherings or isolated areas. In addition, learners mentioned that rape often occurs at night, perpetrated by distant family members when parents are away from alcohol sites in the village.

“*At first, you refuse sex, but later, we buy into it. It is not that we want to get pregnant situations we are in. I have a friend that used to be forced by her cousin's father to have sex. Also, I know a friend who is 16 years old and in an abusive relationship. Her boyfriend forces her to have an abortion.” [Tulela, 18yeras, female]*

#### 4.1.4. Stigma and discrimination and no confidential reporting system for the sexually assaulted victims

Learners reported that fellow learners are the most discriminatory towards them in class, with some teachers also perpetuating discrimination among pregnant and learner parents. The senior female teachers were more supportive than their younger counterparts, who were reported to yell at learners about their pregnancy during class sessions.

“*We cry here. They call us names and mock us, saying things like ‘Oya lya omaandi yeengumi' (they were fed, and their stomach is full of condom lubricant) and ‘ota kuti wala omuvali namwene, inaapanda ku longelwa mumwe naavali' (you mothers keep quiet! we are not happy to be in classes with you). We are afraid of our class teacher; she is not very friendly.” [Leena, 16years, female]*

Relatedly, when asked why they did not report the sexual coercion incidents, participants expressed choosing to remain silent about incidents of sexual coercion due to fear of shame and humiliation. It is insensitive to have their traumatic experiences discussed openly and publicly by villager residents, with the perpetrator even being named in a pervasive culture of stigma and victim-blaming in their communities.

“*Village people are insensitive to absurdly say ‘hano' (this one) ‘kakwatelwe keenghondo komufita wiimuna mafiku aa' (who was recently raped by the cattle herder) while even mentioning the name of the perpetrator.” [Tuyeni, Female, 19 years]*

#### 4.1.5. The absence of community collaborations, stakeholders' involvement, and parental care

The participants mentioned poor stakeholder involvement in preventing and managing learner pregnancy in school settings, despite the ESPPMLP's directives to partner with stakeholders and line ministries. The interviewed learners reflected that stakeholders outside the classroom are not visible as they do not encounter the Ministry of Gender and Social Welfare conducting mobile sessions in school settings but travel long distances to access them. Similarly, they hardly experience the Ministry of Health and Social Services nurse's adolescent-friendly health services in school settings. The Ministry of Safety and Security, responsible for law enforcement, is also hardly involved in addressing them in school settings. Concerning the NGOs, the learners mentioned Ombetjia Yehinga Organisation and Intra Health as the common NGOs that visit their schools. However, their visits are infrequent, occurring only once or twice a year. Although the activities of NGOs are informative, they often occur after school hours, making it difficult for learners to participate.

Furthermore, the interviewed learners revealed that in their community, learner pregnancies and school dropouts have become so frequent that no one shows concern anymore. Community members seem indifferent towards learners who are not their family or relatives. Shockingly, instead of offering support, they mock, gossip, and blame pregnant learners for their family's shortcomings. This societal attitude makes pregnant learners feel ostracised and cursed, leading them to drop out of school.

“*It is a very normal practise here. We do not get surprised when they do not return any more. I know more than 50 young girls in my community who fell pregnant. We see them at bars and cuca shops, breastfeeding their babies. Our community is not taking any action, even when they encounter pregnant or mother learners who drop out of school. ‘Ngayenya eshi topitipo, ovo ghee veli ohaa miti ashike vashona oshavo iha va endeko', which translates to, ‘When you walk by them, elderly women gossip, it is in the family ties, those are promiscuous; they fall pregnant at a tender age, that is how they are.”' [Tekla, 17years female]*

Some interviewed learners reported that poor parental involvement and negligence contributed to learner pregnancy and school dropout. There is a tendency for parents to spend time at alcohol sites without taking part in their children's day-to-day activities. Besides, some parents are reluctant to engage in practical discussions around sexual and reproductive health issues with their children and refuse to consent to contraceptives at health centres. Additionally, it surfaced that for the re-admission of learner-mothers to school, ESPPMLP requires parents or caregivers to sign a letter as a commitment with the school to take care of the infant. However, the learners reflected that some parents refuse to take over such responsibility, and the school refuses to re-admit them.

#### 4.1.6. No privacy and trust among the teachers

Learners claimed that after sharing their personal stories with teachers, they are often speculated about around the school and community. Counselling sessions are also conducted in unfriendly and insensitive settings, such as under the tree and behind classes, which makes learners reluctant to disclose their problems. The lack of privacy and confidentiality was observed during data collection when a life skills teacher unexpectedly walked into the storeroom during an interview with a learner-parent, highlighting the need for a culture of privacy and confidentiality in schools. “*I wish they could talk to me very confidentially, and nobody will spread it. Now I confide in you, as we exist in separate environments. Any mistakes you share your stories here, you would hear from it somewhere else.” [Immanuel, 18-year-old male]*

#### 4.1.7. Age restriction for re-admission for grade nine pregnant and learners-parents after the 1-year maternity leave

It has emerged in the study that despite the ESPPMLP allowing for a 1 year leave of absence for learner-mothers, those over 18 years old are not permitted to return to school after their leave as they are over-aged. Additionally, even if school authorities know that a particular learner-mother's academic progress was disrupted due to maternity leave, they still refuse to admit her. “*That grade 9 age policy prevents many learner-mothers from returning to school. If I become pregnant in grade 9 at 18 or above and take a one-year leave of absence, I will be 19 years or older upon my return, and schools will not enrol me in grade 9 due to my age. The age limit for grade 9 needs to be revised. Why am I only allowed to return to school if I am under 18? What if I started school late or was over 18 when I got pregnant? It is unfair that the government excludes them because of age.” [Tuyoleni 18 years, female]*

#### 4.1.8. Long school holidays

The interviewed learners reasoned that pregnancy occurs during school holidays and semester breaks when they are not restricted to school activities. Thus, they are likely exposed to social evils such as alcohol and drug abuse. Furthermore, learners opined that they quickly forgot about sexual reproductive health education during the holiday. There are also no recreational facilities in the village and seeking relaxation exposes them to activities of unintended pregnancies. In addition, pregnant and learner parents tend to lose school interest and not return after the school holiday.

#### 4.1.9. Walking long distances to school and the lack of accommodation

According to the learners, the long distances between schools in rural areas and the prevalence of savannah areas emerged as significant concerns. Young girls endure 1–2 h walks to and from school during the early morning and late afternoon, thus, possessing some danger of sexual threats and coercions. The pregnant and learner parents cannot endure until their last pregnancy dates. This forces them to drop out of school.

In addition, the study found that many schools in rural communities are scattered, and the junior secondary schools have no hostels. This forces parents to build temporary shacks for their children near the schools. According to the learners, this exposes young learners, some as young as 11, to live alone without parental supervision. As a result, they become vulnerable to sexual exploitation and transactional relationships.

#### 4.1.10. Shortage of flexible facilities for pregnant and learner parents

Besides, the participants cited that pregnant and learner-parents in boarding schools remained in the hostel even after they gave birth. However, it was apparent there was no appropriate furniture for pregnant learners in classrooms, no childcare or caregiving facilities on non-boarding school premises, and no designated space for breastfeeding. The learner-parents indicated that they arrange with their families or use church buildings or open spaces near their schools during breaks for breastfeeding as their requests for better chairs or storage rooms for breastfeeding were deemed unreasonable by school authorities.

#### 4.1.11. The shortage of comprehensive sexual education and accessibility to contraceptives

Some learners reported that they only hear about pregnancy during life skills classes, but no comprehensive programs exist to educate them as the content is minimal. Also, some life-skills teachers offer CSE in bits and pieces, withholding in-depth information because they have their relatives or biological children in their classes. Also, teachers focus on abstaining from sex to avoid getting pregnant without emphasising education on contraceptives. Besides, learners have expressed that only the school's principals and life skills teachers seem to care about learners getting pregnant, as other teachers do not address the issue, making it seem like preventing pregnancy is not a priority.

Participants voiced an objection to walking long distances to access condoms. They reported being constrained from using condoms as the only means of contraception since other forms require parental consent. Learners also mentioned that schools hide condoms in staffrooms, making accessing them freely complex and not private and confidential. “*The only means of contraception here are condoms. Like those ejections[sic], they cannot be used because my parents will not approve. Taking ejection[sic] will decrease fertility.” [Monika, 17 years, female]*.

#### 4.1.12. Exclusion of male learners in CSE programs

In the study, male learners bemoaned not being included in CSE and counselling programs. The visiting NGO and school meetings only focus on educating and supporting girls while insisting they should wait. Even though the ESPPMLP direct that counselling and support for father learners, the male learners reflected that it is provided to pregnant girls and female learners, neglecting them. “*Our school does not involve us in support and counselling sessions, even if we impregnate a girl from another school or the same. I was never summoned to the office about being a learner-father, and I am unaware of how they learned about it.” [Tom, 19 years, male]*

#### 4.1.13. Peer pressure and ignorance

Besides, some learners noted that girls are prone to peer pressure and influence from their male counterparts in exchange for material items and “nice things.” Some learners described their peers as disobedient and ignorant, not listening to their parent's advice. “*The girls at our school like to show off and are attracted to money and alcohol. If a boy offers to give them money daily, the girls will follow them even if they initially decline. A friend wanted money for a farewell party but could not get it from her parents. She went to a man and received N$ 150, but he demanded sex in return.” [Tulonga, female, 17 years]*

### 4.2. Learner-innovative approaches to overcome pregnancy-related school dropout

This theme's central idea was to gather learners' insights on the most effective ways to reduce the incidence of learner pregnancies and enhance learner-parents completing their education. The interventions proposed were derived from the data obtained from the participants and aligned with the directives of the ESPPMLP.

#### 4.2.1. Introduce harsh punishments for learner pregnancy offenders and targeted awareness campaigns for girls and cattle herders

The female learners felt that men, including young boys and older men impregnating school learners, should be held accountable for their actions. Thus, urging the government to impose severe punishments on individuals who impregnate and engage in sexual relationships with young learners. Participants believed that holding the offenders accountable may discourage them and protect vulnerable individuals from exploitation and harm. “*Why is it that when a girl gets pregnant, she is the only one who must drop out of school while the boy continues his education as if nothing happened? Both parties are equally responsible, but only one bears the burden. It is as if I did this alone.” [Peya, 18 years, female]*

Also, the participant felt sensitising girls about the risks of interacting with cattle herders. The young girls must be alerted to the dangers of being alone with strangers, especially with men who may have a different cultural background and may not share the same values and norms. They further urged organised teachings with girls to learn how to detect the signs of manipulation, set boundaries, seek help if uncomfortable or threatened, and advise them not to walk alone in isolated areas after school and avoid late walks. The participant emphasised that the government or community members must engage cattle herders to stop interacting and engaging in sexual activity with girls.

#### 4.2.2. Ban learners from entering “cuca shops and shebeen” and educate the owners

The participants proposed an intervention to prohibit learners from accessing cuca shops and shebeens to reduce the likelihood of engaging in risky behaviours, such as underage drinking and unprotected sex, which causes unintended pregnancies.

#### 4.2.3. Address bullying, stigma, and discrimination in school settings

According to the interviewed learners, to reduce stigma and discrimination in school settings, steps must be taken to hold frequent discussions and ongoing advocacy about its harmful effects and focus on promoting acceptance, respect, and empathy to create a safe and inclusive learning environment. Furthermore, appointing learners' ambassadors and class captains who can advocate against stigma towards pregnant and parenting learners and who may report incidents to the principal for appropriate disciplinary actions. “*If we could have two Learner Representative councils (LRSs) in a class and two class Captains, one sit in front, and the other sits at the back of the corner, where they monitor the situation and report them to the principal for punishment.” [Maya, 17years, female]*

#### 4.2.4. Conduct regular school visits by police officers, social workers, and nurses

Learners suggested regular visits from police officers, social workers, and nurses' schools' visits. Participants believed that police officers' school visits could promote reporting of sexual harassment and educate learners on recognising sexual coercion and harassment. Social workers can provide extensive counselling sessions and identify learners at risk of dropping out of school due to pregnancy through a friendly and confidential system. At the same time, nurses can address health concerns and provide sexual health education to learners.

“*Suppose social workers and professional counsellors can be allocated in schools or could do mobile counselling sessions. Our schools are in rural areas, where such services are far away, and we need a platform to raise pertinent issues. Offloading issues to a stranger is more comfortable than someone you see daily within school premises.” [Immanuel, 18 years Male]*

#### 4.2.5. Reinforce stakeholder collaborations and reinforce parenting

Some participants proposed hosting community meetings involving all parties concerned, including the school administration, parents, community leaders, and Ministry of Education officials, to address the pregnancy-related issue of school dropouts. The learners felt that such meetings might provide a platform for discussing the reasons behind the high dropout rate, brainstorming solutions, and educating the community on the importance of education for learners and their children. They also suggested that learners must be given inclusive and non-judgmental to air their voices to the community members for support during the meeting.

“*I think we need to look for people in the community, nurses, and doctors to talk to girls and boys about the risks and consequences of learner pregnancy. There seems to be insufficient education on this topic, and I believe school principals should talk to us more about it.” [Maya, 15, Female]*

In addition, participants suggested that parents are the most supporting system to influence them to return to school after falling pregnant. Thus, it is crucial to educate them about the importance of involvement in their children's lives beyond attending parent meetings and participating in more school activities. “*Charity starts at home, so the courage to return to school comes from home before anyone else. If your parents do not give you the courage, forget to do it yourself; the teachers counselling and support cannot keep you in school, but parents' assurance.” [John, 18 years, Male]*

#### 4.2.6. Sensitive teachers on confidentiality and sensitivity and introduce a rural-school-friendly anonymous reporting mechanism

Participants cautioned that teachers must be sensitised on handling sensitive information, maintaining confidentiality, and promoting ethical practises.

#### 4.2.7. Remove age restriction for re-entering pregnant and learner parents in Grade Nine

Regardless of exceeding the typical age, participants felt that schools consider extending the opportunity for learner-mothers who took maternity leave at or after the age of 18 to re-enrol for grade 9 full-time and recognise that their pregnancy contributed to their age surpassing the limit.

#### 4.2.8. Reduce or introduce activities during the school holiday

The participants felt that specific activities could be introduced to keep them busy during the long school holidays to prevent boredom. Learners also felt that some home environments may not be conducive to healthy and safe behaviour and could be vulnerable to social evils such as sexual exploitation and abuse. The participants suggested reducing the length of school holidays or organising extra-curricular activities that promote positive behaviours and values among learners.

#### 4.2.9. Promote school hostels create a comprehensive database for the pregnant and learner-parents

Participants believed that providing accommodation in school hostels could keep learners within the safety of the school premises, reducing exposure to shebeens on their way home, where they may be exposed to alcohol, and for them to focus on their studies without distractions. Besides, the interviewed learners suggested that relatives and friends, including the life skills teachers, do home visits to learn more about their challenges and encourage pregnant and learner parents to return to school. “*Their names should be listed in some papers, where teachers could screen them on reasons and engage the parents and the community.” [Tulonga, 14 females, years]*

#### 4.2.10. Expand access to CSE in schools and condom distribution

Learners voiced that condoms are expensive to buy from shops, and some must travel long distances to clinics to access them. Thus, they found that schools must distribute condoms to learners in school settings to reduce the risk of unintended pregnancies and the spread of sexually transmitted infections. “*Condoms are expensive to purchase at local shops. We buy a pack of three condoms for $20 at a nearby service station. We spend little cash on transportation to the clinic over 20 kilometres from our school. Schools must provide easier access to condoms and other contraception.” [Taya 17 years male]*

#### 4.2.11. Ongoing advocacy

Participants proposed schools, particularly life skills teachers, to continuously remind learners and speak about pregnancy, not only when they see learners leaving school.

“*Ova pumbwa kupoya manga omeya ina aya mumwe nomandu. Direct translation ‘Talk before water is mixed with sand. Meaning they must consistently advocate for prevention before the situation worsens.”' [Taati, 17 years old Female]*

## 5. The findings and discussions

This study aimed to investigate the perspectives of learners regarding the prevention and management of learner pregnancy and school dropout, specifically concerning the ESPPMLP. Table 1 provide a summary of the sample of the study participants by educational circuits, schools, participants, and gender. As shown in Table 2, two main themes emerged from the study: learner perspectives on the causes of pregnancy and school dropout and learner-focused approaches to addressing these issues. The learner perspectives on the causes of pregnancy and school dropout findings were further categorised into four: community hostility, educational factors, lack of infrastructure and resources, and learner unawareness. The learner-focused approaches were categorised into four initiatives: mitigate community hostility, improve educational conditions, allocate necessary infrastructure and resources, and raise awareness among learners.

**Table 1:** Summary of the sample of the study participants by educational circuits, schools, participants, and gender.

**Educational circuits**	**Rural school**	**School level**	**Male participants**	**Female participants**	**Mother learners**	**Father-learners**	**Pregnant learners**
One	School A	Primary	2	4			
Two	School B	Primary		4			
Three	School C	Junior secondary	1	3	3	1	
Four	School D	Junior secondary	2	3	2		1
Five	School E	Combined	2	3	2		1
Six	School F	Junior secondary	1	4	2		
Seven	School G	Combined	2	4	2		
Eight	School H	Secondary	2	2	3		
Nine	School I	Secondary	1	3			
Ten	School J	Combined	2	1			
Total number of participants: 63							

**Table 2:** Emerging themes.

**Perspectives of learners on causes of learner pregnancy and school dropout**		**Learner-focused approaches**	
Community hostility	Elder men, including cattle herders and police officers, preying on young girls	Mitigate community hostility	Introduce harsh punishments for learner pregnancy offenders and create awareness for girls and the cattle herders
	The threat of sexual coercion		
	The proximity of alcohol sites “Sheeben and Cuca Shop” within schools		Ban learners from entering Cuca Shops and Shebeen and educate the owners of the alcohol sites about it
	Stigma and discrimination		Address bullying, stigma, and discrimination in school settings
	The lack of community collaborations, stakeholders involvement, and parental care		Reinforce stakeholder collaboration, community partnership, and parental care
Educational factors	Lack of trust among the teachers		Sensitive and train teachers on confidentiality, ethics, and sensitivity
	No confidential reporting system for the sexually assaulted victim	Improve educational conditions	Introduce a flexible and anonymous reporting system
	Age restriction for re-admission for grade nine pregnant and learners after the 1-year maternity leave		Remove age restriction for re-entering pregnant and learner parents in grade nine
	Long school holidays and walking long distances to school		Reduce school holiday Create a database for pregnant and learner parents' school dropout
The lack of infrastructure and resources	Unfriendly facilities for expectant and learner parent	Allocate necessary infrastructure and resources	Expand access to CSE in schools and condom distribution
	The lack of sexual education and inaccessibility to contraceptives		
	Exclusion of male learners in CSE programs		
Learner unawareness	Peer pressure and ignorance	Raise awareness among learners with ongoing advocacy	Ongoing advocacy

### 5.1. Learners' perspectives on the causes of learner pregnancy and school dropout

As per the perspectives of learners, the causes of learner pregnancy and school dropout are influenced by community hostility or unwelcoming behaviour that tolerates or promotes harmful attitudes with factors such as elder men, cattle herders, and police officers preying on young girls into sexual affairs, the proximity of alcohol sites “shebeen and cuca shop” within schools premises, the threat of sexual coercion, stigma and discrimination, the lack of community collaborations, stakeholders involvement and parental care, a lack of trust among the teachers, and the lack of confidential reporting system for the sexually assaulted victims.

Relating to the implementation of the ESPPMLP that only prohibits and punishes schoolteachers who engage in sexual relationships with learners, the study revealed that there is a trend of older men in communities such as cattle herders, police officers, and prominent and respected men whose names are concealed who bribes, prey, and entice learners, particularly females, against their consent, leading to learner pregnancies and school dropout. Those men abuse power and exploit vulnerable young girls, eroding the sense of safety and security while creating an atmosphere of fear and distrust within the community. Our findings correlate with the Namibian media outlets that widely reported that older men engage with school-going girls (The Namibian, 2022a; Namibian Sun, 2023; New Era, 2023). In contrast to media reports of male teachers bribing parents for their silence in Namibian schools (Pearson and Van Der Berg, 2015; UNESCO, 2018), this study also established that many learner pregnancies are not caused by teachers but rather by someone outside the school system (Nekongo-Nielsen and Mbukusa, 2013). As the scope was limited to exclusively school-going learners, we could not verify these claims by consulting with other members of the communities.

Furthermore, this study established that the proximity of alcohol sites to school premises exposes learners to alcohol at an early age, stimulates underage drinking, and potentially increases the chances of earlier sexual debut, and loss of interest in school attendance. Pearson and Van Der Berg (2015) noted similar results with drunkenness and fighting in the proximity of schools in Namibia. The study shows that while the ESPPMLP promotes schools as safe environments free from sexual abuse and harassment (Ministry of Education, 2012), participative learners endure the threat of sexual coercion and increased likelihood of sexual violence and harassment. Furthermore, walking long distances to and from school triggers and expose learners to sexual relationship, earlier pregnancy, and dropping out of school. Pearson and Van Der Berg (2015) and Burton et al. (2011) also highlighted the risks of youth victimisation and the challenges of attending school when walking long distances in Namibia. In addition, while the previous study insinuated Namibian schools as sites of sexual harassment (Legal Assistance Centre, 2017; Burton et al., 2011; UNESCO, 2018), we recognise that the safety and wellbeing of learners are not only influenced by school environments but also by the broader community where they live.

Despite the provisions set forth by the ESPPMLP to combat stigma and discrimination against pregnant or learner-parents (Ministry of Education, 2012), the study revealed a culture of stigma and discrimination in school settings that prevail among learners and junior teachers, which intensify a hostile environment for pregnant and learners' parents to drop out of school. This finding is in accordance with the previous studies in Namibia by Burton et al. (2011), Nekongo-Nielsen and Mbukusa (2013), Pearson and Van Der Berg (2015), Legal Assistance Centre (2017), and UNESCO (2018). This study further revealed the attitudes of community hostility towards pregnant and learner parents that relate learner pregnancy to family failures and generational curses, leading to increased school dropout rates. The stigma and discrimination are also propagated upon learners who have experienced sexual assault through victim-blaming, shaming, and humiliation. While the ESPPMLP promotes the counselling and support session and emphasises confidentiality in school settings (Ministry of Education, 2012), the absence of flexible and confidential reporting mechanisms and the lack of trust among teachers intensifies learner pregnancy and school dropout, hinders the reporting process, and obstructs the provision of essential support to those in need.

Additionally, while the ESPPMLP focuses on stakeholder sharing responsibility, learners in the study claimed that they encounter inadequate stakeholder support as there are no formal linkages between government institutions (the three ministerial bodies) and schools for effective communication, thus causing cancellations and delays in learner referrals and interventions (UNESCO, 2017). The learners in the study also claimed that stakeholders outside the classroom were invisible, particularly police officers, nurses, social workers, and parents, with only a few NGOs visiting the school occasionally. The study established that learners travel long distances to nearby towns for services, such as access to social workers for counselling and access to contraceptives, which can be time-consuming and inconvenient, leaving them without the necessary support and finally making them to give up. In addition, it emerged that the community members do not care about supporting or encouraging learners and pregnant learners who are not their relatives. UNESCO (2018) highlighted similar findings, highlighting the absence of formal connexions between schools, health services, and relevant stakeholders such as parents, government institutions, and NGOs in Namibia. These circumstances increase the likelihood of learner pregnancy and school dropout.

Considering educational factors, the age restriction to re-enrollment in grade nine for the learner-mothers above 18 years after 1 year of maternity leave, and the long school holiday emerged in this study. While the ESPPMLP directs that learner-mothers may choose to return to school or extend their leave to 1 year, learner-parents, particularly mothers over 18 years, struggle to seek admission into the formal system. The Namibia Ministry of Education has repetition policies restricting learners over 18 years from sitting twice in grade nine (Pearson and Van Der Berg, 2015). This applies even to learner-parents over 18 upon the return of their 1 year leave of absence as schools refuse to admit them as they are categorised as over-aged for grade nine. This restriction disregards the importance of providing educational support and opportunities for learner-mothers, potentially leading to higher dropout rates among this group. Pearson and Van Der Berg (2015) also highlighted the age limit to school enrollment for over 16,000 Namibian children who drop out of school every year after failing grade 10 (10). Regarding long school holidays, this study found that long school holidays could contribute to learner pregnancy and dropout as participants believed that extended breaks from school provide free time for young individuals, which may increase the likelihood of engaging in risky behaviours, including sexual activity and alcohol abuse.

Insufficient infrastructure and the lack of resources were also highlighted in this study, including the walking long distances to school, lack of accommodation and recreational facilities, unfriendly facilities for pregnant and learner parents, the lack of sexual education and inaccessibility to contraceptives, and the exclusion of males into sexual reproduction health programs. The study noted that schools in rural Namibia areas are distanced (Pearson and Van Der Berg, 2015). Thus, the learners in this study experience walking to and from school in isolated rural savannah areas while exposed to sexual harassment. Previous literature highlighted the risks of youth victimisation by attending school when walking long distances in Namibia (Burton et al., 2011; Pearson and Van Der Berg, 2015). The lack of recreational facilities in rural areas results in learners having too much free time and fewer structured activities. Adekola and Mavhandu-Mudzusi (2023) found similar results on the lack of recreational facilities and risky sexual behaviours.

Regarding the directives of the ESPPMLP that emphasise the provision of CSE, the learners in this study encountered a shortage of CSE in their schools and no access to contraception as the ESPPMLP strictly instructs school principals only to provide contraceptive information but not to distribute condoms in school settings. Our findings contrast with UNESCO (2018), which reports limited access to contraceptives in schools due to resistance from school principals, teachers, parents, and community sensitivities. In addition, the male learners in the study are not being catered to within CSE programs, neither offered counselling nor support for impregnating fellow learners. This constraining of contraceptives in school settings is likely to lead to a lack of knowledge and awareness about safe sex practises and, unprotected sex, risky sexual behaviours. Male exclusion may point to a lack of gender insensitivity and awareness. This is a common phenomenon in many sub-Saharan countries, as highlighted by various scholars, such as Nyariro (2018); Mutua et al. (2019), Zulu et al. (2019), Mogotsi and Mwetulundila (2020), Ramalepa et al. (2020), Chinkondenji (2022), Ruzibiza (2021), and Adekola and Mavhandu-Mudzusi (2023), who raised similar concerns.

The inadequate restroom facilities, lactating rooms, and insufficient support for breastfeeding create an unfriendly school atmosphere that discourages pregnant and learner-parents from continuing their education, leading to early school dropout. Pearson and Van Der Berg (2015) emphasised the health hazards of the absence of proper sanitation facilities, contributing to pregnant learners dropping out of school. In the study, the absence of adequate accommodation and hostels for young learners, some as young as 11 years old, in makeshift arrangements exposes them to pregnancy and school dropout potential risks.

The issue of learner unconsciousness linked to peer pressure and ignorance among learners who engage in sexual and transactional relationships for financial support and access to free alcohol and entertainment was further noted. Previous studies in sub-Saharan African countries also identified peer pressure and ignorance as critical barriers to preventing learner pregnancy (UNESCO, 2018; Ramalepa et al., 2021; Ruzibiza, 2021). The study agrees with Adekola and Mavhandu-Mudzusi (2023), who emphasised that despite the sexuality education programs, young people might not apply acquired sexuality due to peer pressure and ignorance.

### 5.2. Learner-focused approaches to preventing and managing learner pregnancy and school dropout

This study revealed learner-focused approaches to prevent learner pregnancy and school dropout, highlighting the need for interventions that mitigate community hostility, improve educational conditions, allocate necessary infrastructure and resources, and execute ongoing advocacy. The identified approaches aim to improve the implementation of the ESPPMLP and ultimately reduce the rate of learner pregnancy and school dropout in rural Namibia schools.

Mitigating the community hostility includes the following: introducing harsh punishments for learner pregnancy and offenders and creating awareness among girls and cattle herders; prohibiting learners from entering cuca shops and shebeen and educating the owners; addressing, enhancing stakeholder collaboration, and community partnership and parenting; bullying, stigma, and discrimination in school settings; sensitising teachers on confidentiality; and introducing a rural-school-friendly anonymous reporting mechanism.

Introducing strict legal repercussions and punishment for learner pregnancy and enforcing severe consequences may hold perpetrators accountable. The fear of legal repercussions may discourage older men, cattle herders, police officers, and taxi drivers from engaging in unlawful relationships with learners and shift the focus from blaming the learners to holding the adults responsible. This may also send a message that such actions are unacceptable. Nekongo-Nielsen and Mbukusa (2013) stress that schools should record the perpetrators' identities to track and bring them to book.

Educating learners, cattle herders, and older men about the legal risks associated with interacting with minors may increase awareness about statutory rape and encourage minors to speak up when feeling unsafe. This sensitisation can also educate the cattle herders about the importance of girls' education while empowering vulnerable learners to protect themselves and report incidents of abuse. Studies established that involving perpetrators in developing strategies and solutions has decreased learner pregnancy cases (Nekongo-Nielsen and Mbukusa, 2013). Prohibiting learners from accessing alcohol sites may protect them from engaging in risky behaviours. Combining this measure with awareness campaigns that educate learners on the dangers of alcohol consumption while targeting alcohol site owners located near schools may have positive outcomes in preventing learner pregnancy and school dropout rates.

Reinforcing collaboration among stakeholders, including community leaders, educators, and government officials, may achieve significant results in addressing these challenges. Establishing awareness and guidelines to monitor and report inappropriate behaviour for girls in the community is necessary. Empowering and reinforcing parenting through community leadership can help increase parental involvement in education. This can be achieved through information-sharing platforms and meetings, capacity building, resource mobilisation, and monitoring activities with local chiefs. One school community in Ghana successfully used this approach, and its learner pregnancy and school dropout rate decreased from seven in 2015 to three in 2016 and none in 2017 (World Vision, 2020). Adekola and Mavhandu-Mudzusi (2023) cautioned that it could address parental objections, misconceptions, and contradictory messages about sexuality education. Organising regular mobile counselling sessions in school settings and mobile clinics in rural schools may identify learners at risk of pregnancy or dropping out of school, provide them with counselling, and build confidence to continue their education. Law enforcement visits may deter perpetrators from targeting girls in the community, as they will be held accountable for their actions.

Addressing the bullying, stigma, and discrimination is essential to creating a safe and accepting culture in the community to support pregnant and learner-parents. Nekongo-Nielsen and Mbukusa (2013) also emphasise establishing a secure, friendly, and encouraging learning environment. This could be correctly implemented when teachers and community members are trained on confidentiality, gender sensitivity, care, and the ESPPMLP mandate. Studies recommended that knowledge about the re-entry policies may influence the outcomes of learner pregnancy in school settings (Zuilkowski et al., 2019). Besides, establishing an anonymous reporting mechanism for sexual violence at school and community levels may provide survivors with a safe and supportive environment to report incidents of sexual assaults. This can break down the culture of shame, silence, and victim blaming perpetuated in schools and communities. World Vision (2020) and UNESCO (2021) emphasise that these systems can successfully enable learners to report their concerns without revealing their identities.

Concerning the educational intervention, the study recommends the amendment of the school repetition policy to accommodate learner-mothers who may have turned 18 years or older during maternity leave and raise awareness among school authorities about learner-mothers' right to education. Removing this restriction can make the ESPPMLP inclusive to learner-parents. Furthermore, creating a database for pregnant and learner parents' school dropouts to follow up with parents and relevant stakeholders on their challenges and whereabouts for interventions to return to schools. The Namibian EMIS report does not report the return rates of learner pregnancy and school dropout (Ministry of Education, 2021). Studies emphasised that by providing statistics on the number of pregnancies caused by school dropouts, the completion and admission rates determine whether policies have been successful (Kennedy, 2017).

The allocation of infrastructure and resources, such as the provision of accommodation, can be enhanced by providing school hostels, which can protect learners from social issues in their villages, reduce long commutes to school, and discourage risky behaviours after school, thus promoting academic focus. These findings correspond to those of Pearson and Van Der Berg (2015), who viewed hostels as a means of alleviating poverty and overcoming physical distance. Additionally, reducing long school holidays and providing recreational activities through community affiliations can shorten learners' idle time and reduce their inclination to engage in risky activities. Expanding CSE and distributing condoms in schools may empower learners to make informed decisions about their sexual health, preventing unintended pregnancies and enabling them to continue their education. This can be achieved by increasing accessibility to contraceptives and sexual and reproductive health services through mobile clinics in school settings and the community (Lukolo and van Dyk, 2014; UNESCO, 2017). This must be accompanied by ongoing advocacy efforts that may prioritise the issue and remind learners of the consequences of learner pregnancy and school dropout.

In addition to the learner's proposed interventions, various countries have implemented strategies to prevent and manage learner pregnancy and school dropout, including nurseries and childhood centres near schools in Gabon, special accommodations for young mothers in Cape Verde and Senegal, and conditional cash transfers, as demonstrated in Colombia (World Vision, 2020; Maharaj, 2022). Finally, this study determined that addressing the root causes of risky behaviours among learners is crucial for creating sustainable solutions to preventing learner pregnancy and school dropout.

## 6. Conclusion

The findings of this study indicate learners' perspectives on various factors that contribute to learner pregnancy and dropout in rural Namibian schools, such as older men and cattle herders preying on young girls, peer pressure, ignorance and stigma, long-distance walking to school, threats of sexual coercion, poor reporting systems, long school holidays, lack of recreational facilities, limited access to CSE and contraceptives, unfriendly school and community environments for pregnant learners and learner mothers, limited stakeholder involvement, and age restrictions on re-admission. Regarding the ESPPMLP, this study proposed learner-focused approaches such as prohibiting learners' access to cuca shops and shebeens, implementing harsh punishments for offenders involved in learner pregnancy and sexual engagement, conducting targeted awareness campaigns for girls and cattle herders, organising regular visits by nurses, social workers, and police officers to schools, addressing bullying and stigma, fostering a culture of acceptance in school settings, empowering parenting, strengthening stakeholder collaborations, reducing long school holidays, establishing a comprehensive database, expanding access to CSE and condom distribution, and ongoing advocacy efforts.

This study also emphasises that policymakers must incorporate learners' perspectives into interventions targeting learners' pregnancy interventions, such as mitigating community hostility and behaviours, improving educational conditions, allocating necessary infrastructure and resources, and raising awareness among learners about the consequences of learners' pregnancies and dropping out of school. Future research should explore the viewpoints of teachers, parents, and relevant stakeholders and investigate the regulations governing alcohol sites near schools.

## 7. Limitations

The finding of this study was based on a small population and cannot be generalised to all rural schools in Namibia. The lack of out-of-school pregnant and learner-parents' perspectives and the inclusion of learners as old as 23 years attending schools within the 13–18 age range may influence the overall findings and generalisability of the study. Learners may have felt anxious or uncomfortable sharing their perspectives with the researcher, who had no prior relationship with them. Additionally, limited data from the stakeholders, such as the Namibia Ministry of Gender and Equality, the Ministry of Safety and Security, and the Ministry of Health and Social Services, may create further challenges in concluding.

## Data availability statement

The raw data supporting the conclusions of this article will be made available by the authors, without undue reservation.

## Ethics statement

The studies involving human participants were reviewed and approved by the Namibia Ministry of Education. Written informed consent to participate in this study was provided by the participants' legal guardian/next of kin.

## Author contributions

The author confirms being the sole contributor of this work and has approved it for publication.
